# Supplementary material for: NMDA receptors in visual cortex are necessary for normal visuomotor integration and skill learning
Source: eLife. 2022 Feb 16;11:e71476. doi: 10.7554/eLife.71476 (PMC8901170; doi:10.7554/eLife.71476)
Supplement: Supplementary file 1. — (A) Table of all statistical information for all figure panels that display the results of statistical testing. (B) List of the number of mice of each genotype for each experiment. [file elife-71476-supp1.docx]

Supplementary File 1A. Statistics

All values are rounded to two significant digits, except values smaller than 10_­_^-5^. The tests used were a two-sample rank sum test (ranksum), two-sample independent t-test (t-test 2), one sample t-test (t-test 1), or a bootstrap estimate of the 95% confidence interval (CI). Sample sizes were estimated based on previous experiments performed in the same behavioral paradigms (Attinger et al., 2017; Heindorf et al., 2018).

| Reference | Description | Test | N_1_ (mice) | N_2_ (mice) | Unit | P-value/CI |  |
| --- | --- | --- | --- | --- | --- | --- | --- |
| Figure 1F | Control (N_1_) vs ΔGrin1_juv_ (N_2_) | t-test 2 | 10 (2) | 10 (2) | Sections | <10^-5^ |  |
| Figure 1G | Control (N_1_) vs ΔGrin1_juv_ (N_2_) |  |  |  |  |  |  |
|  | L2/3 | ranksum | 918 (2) | 874 (2) | Nuclei | <10^-5^ |  |
|  | L4 | ranksum | 1272 (2) | 839 (2) | Nuclei | <10^-5^ |  |
|  | L5 | ranksum | 954 (2) | 1570 (2) | Nuclei | <10^-5^ |  |
|  | L6 | ranksum | 1442 (2) | 1718 (2) | Nuclei | <10^-5^ |  |
|  | PV | ranksum | 350 (2) | 307 (2) | Nuclei | <10^-5^ |  |
|  | SST | ranksum | 189 (2) | 157 (2) | Nuclei | <10^-5^ |  |
|  | VIP | ranksum | 265 (2) | 122 (2) | Nuclei | 0.60 |  |
| Figures 2A-2C | Control (N_1_) vs ΔGrin1_juv_ (N_2_) |  |  |  |  |  |  |
|  | Mismatch response | t-test 2 | 794 (5) | 551 (5) | Neurons | 0.000082 |  |
|  | Drifting grating response | t-test 2 | 794 (5) | 551 (5) | Neurons | 0.030 |  |
|  | Running onset response (closed-loop) | t-test 2 | 794 (5) | 551 (5) | Neurons | 0.054 |  |
| Figure 2D | Control (N_1_) vs ΔGrin1_juv_ (N_2_) | t-test 2 | 2625 (14) | 1986 (16) | Neurons | 0.81 |  |
| Figure 2F | Control (N_1_) vs ΔGrin1_juv_ (N_2_) | t-test 2 | 2625 (14) | 1986 (16) | Neurons | <10^-5^ |  |
| Figure 2-figure supplement 1A | CT (N_1_) vs ΔGrin1_juv_ control (N_2_) | t-test 2 | 2259 (9) | 2080 (12) | Neurons | 0.14 | |
|  | NT (N_1_) vs ΔGrin1_juv_ (N_2_) | t-test 2 | 2103 (9) | 1516 (14) | Neurons | 0.57 | |
| Figure 2-figure supplement 1B  (Left) | CT | CI | 10^4^ | N/A | PCA angles | [-34.36, -4.16] | |
|  | NT | CI | 10^4^ | N/A | PCA angles | [ 29.63, 43.40] | |
| Figure 2-figure supplement 1B  (Right) | ΔGrin1_juv_ control hemisphere | CI | 10^4^ | N/A | PCA angles | [-50.73, -13.77] | |
|  | ΔGrin1_juv_ | CI | 10^4^ | N/A | PCA angles | [-10.66, 10.90] | |
| Figure 2-figure supplement 1C | Pre (N_1_) vs post (N_2_) MK-801 | t-test 2 | 2443 (9) | 2443 (9) | Neurons | <10^-5^ | |
| Figure 2-figure supplement 1D | Pre (N_1_) vs post (N_2_) MK-801 | t-test 2 | 2443 (9) | 2443 (9) | Neurons | <10^-5^ | |
| Figures 3A-C | ΔGrin1_adult_ (N_2_) vs control (N_1_) |  |  |  |  |  |  |
|  | Mismatch response | t-test 2 | 912 (9) | 1184 (11) | Neurons | 0.45 |  |
|  | Drifting grating response | t-test 2 | 912 (9) | 1184 (11) | Neurons | 0.57 |  |
|  | Running onset response (closed-loop) | t-test 2 | 912 (9) | 1184 (11) | Neurons | 0.45 |  |
| Figure 3D | Control (N_1_) vs ΔGrin1_adult_ (N_2_) | t-test 2 | 1281 (11) | 1547 (13) | Neurons | <10^-5^ |  |
| Figure 3F | Control (N_1_) vs ΔGrin1_adult_ (N_2_) | t-test 2 | 1281 (11) | 1547 (13) | Neurons | <10^-5^ |  |
| Figure 4C | Control_adult_ (N_1_) vs |  |  |  |  |  |  |
|  | ΔGrin1_juv_ control (N_2_) | t-test 2 | 869 (10) | 794 (5) | Neurons | 0.15 |  |
|  | ΔGrin1_juv_ (N_2_) | t-test 2 | 869 (10) | 551 (5) | Neurons | 0.000038 |  |
|  | ΔGrin1_adult_ control (N_2_) | t-test 2 | 869 (10) | 912 (9) | Neurons | 0.37 |  |
|  | ΔGrin1_adult_ (N_2_) | t-test 2 | 869 (10) | 986 (10) | Neurons | 0.24 |  |
| Figure 5C  (Days) | ΔGrin1_juv_ avg. performance index, days 1-2 vs 6-7 | t-test 1 | 6 | 6 | Mice | 0.22 |  |
|  | ΔGrin1_adult_ avg. performance index, days 1-2 vs 6-7 | t-test 1 | 13 | 13 | Mice | 0.0002 |  |
|  | Control_juv_ avg. performance index, days 1-2 vs 6-7 | t-test 1 | 6 | 6 | Mice | 0.0037 |  |
| Figure 5C  (Groups) | Average performance index on days 6-7 |  |  |  |  |  |  |
|  | Control_juv_ (N_1_) vs ΔGrin1_juv_ (N_2_) | t-test 2 | 6 | 6 | Mice | 0.0013 |  |
|  | Control_juv_ (N_1_) vs ΔGrin1_adult_ (N_2_) | t-test 2 | 6 | 13 | Mice | 0.39 |  |
|  | ΔGrin1_juv_ (N_1_) vs ΔGrin1_adult_ (N_2_) | t-test 2 | 6 | 13 | Mice | 0.016 |  |
| Figure 5E | Control_juv_ (N_1_) vs ΔGrin1_juv_ (N_2_) | t-test 2 | 6 | 6 | Mice | 0.0098 |  |
|  | Control_juv_ (N_1_) vs ΔGrin1_adult_ (N_2_) | t-test 2 | 6 | 13 | Mice | 0.30 |  |
|  | ΔGrin1_juv_ (N_1_) vs ΔGrin1_adult_ (N_2_) | t-test 2 | 6 | 13 | Mice | 0.0073 |  |
|  | Control_juv_  (N_1_) vs 0 | t-test 1 | 6 | N/A | Mice | 0.0057 |  |
|  | ΔGrin1_juv_ (N_1_) vs 0 | t-test 1 | 6 | N/A | Mice | 0.91 |  |
|  | ΔGrin1_adult_ (N_1_) vs 0 | t-test 1 | 13 | N/A | Mice | 0.0019 |  |
| Figures 6C-6E | Control (N_1_) vs paAIP2_CaMKIIα_ (N_2_) |  |  |  |  |  |  |
|  | Mismatch response | t-test 2 | 781 (5) | 928 (5) | Neurons | <10^-5^ |  |
|  | Drifting grating response | t-test 2 | 781 (5) | 928 (5) | Neurons | <10^-5^ |  |
|  | Running onset response (closed-loop) | t-test 2 | 781 (5) | 928 (5) | Neurons | 0.0013 |  |
| Figures 6F-6H | Control (N_1_) vs paAIP2_SST_ (N_2_) |  |  |  |  |  |  |
|  | Mismatch response | t-test 2 | 1277 (7) | 807 (5) | Neurons | <10^-5^ |  |
|  | Drifting grating response | t-test 2 | 1277 (7) | 807 (5) | Neurons | <10^-5^ |  |
|  | Running onset response (closed-loop) | t-test 2 | 1277 (7) | 807 (5) | Neurons | <10^-5^ |  |
| Figure 6-figure supplement 1B | Control (N_1_) vs paAIP2_CaMKIIα_ (N_2_) | t-test 2 | 781 (6) | 928 (6) | Neurons | <10^-5^ | |
| Figure 6-figure supplement 2C-E | Control (N_1_) vs paAIP2_PV_ (N_2_) |  |  |  |  |  | |
|  | Mismatch response | t-test 2 | 1256 (5) | 919 (5) | Neurons | <10^-5^ | |
|  | Drifting grating response | t-test 2 | 1256 (5) | 919 (5) | Neurons | <10^-5^ | |
|  | Running onset response (closed-loop) | t-test 2 | 1256 (5) | 919 (5) | Neurons | <10^-5^ | |
| Figure 6-figure supplement 3A-C | paAIP2_CaMKIIα_ day 1 (N_1_) vs paAIP2_CaMKIIα_ day 2 (N_2_) |  |  |  |  |  | |
|  | Mismatch response | t-test 2 | 928 (6) | 926 (6) | Neurons | <10^-5^ | |
|  | Drifting grating response | t-test 2 | 928 (6) | 926 (6) | Neurons | <10^-5^ | |
|  | Running onset response (closed-loop) | t-test 2 | 928 (6) | 926 (6) | Neurons | <10^-5^ | |
| Figure 6-figure supplement 3D-F | paAIP2_SST_ day 1 (N_1_) vs paAIP2_SST_ day 2 (N_2_) |  |  |  |  |  | |
|  | Mismatch response | t-test 2 | 807 (5) | 397 (2) | Neurons | <10^-5^ | |
|  | Drifting grating response | t-test 2 | 807 (5) | 397 (2) | Neurons | <10^-5^ | |
|  | Running onset response (closed-loop) | t-test 2 | 807 (5) | 397 (2) | Neurons | <10^-5^ | |
| Figure 6-figure supplement 3G | Control_adult_ (N_1_) vs |  |  |  |  |  | |
|  | paAIP2_CaMKIIα_ (N_2_) | t-test 2 | 1000 (12) | 926 (6) | Neurons | <10^-5^ | |
|  | paAIP2_SST_ (N_2_) | t-test 2 | 1000 (12) | 397 (2) | Neurons | 0.0044 | |
|  | paAIP2_PV_ (N_2_) | t-test 2 | 1000 (12) | 492 (2) | Neurons | <10^-5^ | |
| Figure 7A | Control_adult_ (N_1_) vs |  |  |  |  |  |  |
| Figure 7A | ΔGrin1_adult_ (N_2_) | t-test 2 | 1000 (12) | 1547 (13) | Neurons | <10^-5^ |  |
|  | ΔGrin1_juv_ (N_2_) | t-test 2 | 1000 (12) | 1516 (14) | Neurons | 0.0032 |  |
|  | paAIP2_CaMKIIα_ (N_2_) | t-test 2 | 1000 (12) | 928 (6) | Neurons | <10^-5^ |  |
|  | paAIP2_SST_ (N_2_) | t-test 2 | 1000 (12) | 1022 (6) | Neurons | <10^-5^ |  |
|  | aAIP2_PV_ (N_2_) | t-test 2 | 1000 (12) | 1705 (8) | Neurons | 0.013 |  |

Supplementary File 1B. Number of mice per experiment

We imaged all experimental mice on both hemispheres whenever possible. The table below lists the number of mice as a function of whether they were imaged in the control (Ctrl) or the intervention (Int.) hemisphere only, or in both hemispheres.

|  | Number of mice | | | |
| --- | --- | --- | --- | --- |
| Dataset | Ctrl only | Int. only | Both | **Total** |
| ΔGrin1_juv_ | 4 | 5 | 10 | **19** |
| ΔGrin1_adult_ | 1 | 3 | 10 | **14** |
| paAIP2_CaMKIIα_ | 0 | 0 | 6 | **6** |
| paAIP2_SST_ | 1 | 0 | 6 | **7** |
| paAIP2_PV_ | 0 | 0 | 6 | **6** |
| MK-801 | 0 | 9 | 0 | **9** |
| Control_adult_ | 12 | 0 | 0 | **12** |

Attinger, A., Wang, B., and Keller, G.B. (2017). Visuomotor Coupling Shapes the Functional Development of Mouse Visual Cortex. Cell *169*, 1291-1302.e14.

Heindorf, M., Arber, S., and Keller, G.B. (2018). Mouse Motor Cortex Coordinates the Behavioral Response to Unpredicted Sensory Feedback. Neuron *99*, 1040-1054.e5.
